# Less-structured time in children's daily lives predicts self-directed executive functioning

**DOI:** 10.3389/fpsyg.2014.00593

**Published:** 2014-06-17

**Authors:** Jane E. Barker, Andrei D. Semenov, Laura Michaelson, Lindsay S. Provan, Hannah R. Snyder, Yuko Munakata

**Affiliations:** ^1^Department of Psychology and Neuroscience, University of Colorado BoulderBoulder, CO, USA; ^2^Department of Psychology, University of DenverDenver, CO, USA

**Keywords:** cognitive development, self-directed executive function, leisure time, unstructured activities, verbal fluency

## Abstract

Executive functions (EFs) in childhood predict important life outcomes. Thus, there is great interest in attempts to improve EFs early in life. Many interventions are led by trained adults, including structured training activities in the lab, and less-structured activities implemented in schools. Such programs have yielded gains in children's externally-driven executive functioning, where they are instructed on what goal-directed actions to carry out and when. However, it is less clear how children's experiences relate to their development of self-directed executive functioning, where they must determine on their own what goal-directed actions to carry out and when. We hypothesized that time spent in less-structured activities would give children opportunities to practice self-directed executive functioning, and lead to benefits. To investigate this possibility, we collected information from parents about their 6–7 year-old children's daily, annual, and typical schedules. We categorized children's activities as “structured” or “less-structured” based on categorization schemes from prior studies on child leisure time use. We assessed children's self-directed executive functioning using a well-established verbal fluency task, in which children generate members of a category and can decide on their own when to switch from one subcategory to another. The more time that children spent in less-structured activities, the better their self-directed executive functioning. The opposite was true of structured activities, which predicted poorer self-directed executive functioning. These relationships were robust (holding across increasingly strict classifications of structured and less-structured time) and specific (time use did not predict externally-driven executive functioning). We discuss implications, caveats, and ways in which potential interpretations can be distinguished in future work, to advance an understanding of this fundamental aspect of growing up.

## Introduction

Why do young children often forget (or outright refuse) to put on a coat before leaving the house on a snowy day? The choice to put on a jacket may seem frustratingly obvious to parents and older siblings, but this simple decision arises from a surprisingly complex interplay of behaviors. Children must keep in mind a goal (staying warm and dry) that is not yet relevant in the comfort of a warm house. They must inhibit the urge to proceed with a regular sequence of tasks (put on socks and shoes and head out the door), and instead modify their routine to include something new (pulling a coat from the closet). Unless someone intervenes, this change in the status quo must be accomplished without any external reminders (a visible coat, or a well-timed reminder from a caregiver).

To accomplish each of these tasks, children must engage executive functions (EFs), the cognitive control processes that regulate thought and action in support of goal-directed behavior. EFs develop dramatically during childhood (e.g., Gathercole et al., 2004; Zelazo et al., 2008; McAuley et al., 2011; Munakata et al., 2012), and support a number of higher-level cognitive processes, including planning and decision-making, maintenance and manipulation of information in memory, inhibition of unwanted thoughts, feelings, and actions, and flexible shifting from one task to another. Researchers have used a variety of laboratory tasks to measure child EFs, including table-top behavioral tasks (e.g., the classic marshmallow test, card-sorting tasks) and computerized tasks (e.g., Go/No-go, Flanker), many of which tap multiple aspects of EF. Over the past decade, EFs have emerged as critical, early predictors of success across a range of important outcomes, including school readiness in preschoolers (Miller et al., 2013), as well as academic performance at school entry (Blair and Razza, 2007; Cameron et al., 2012) and beyond (St Clair-Thompson and Gathercole, 2006; Best et al., 2011). Moreover, children with worse EF go on to have poorer health, wealth, and social outcomes in adulthood than children with better EF, even after controlling for differences in general intelligence (Moffitt et al., 2011).

Given the established links between early EFs and later life outcomes, a number of studies have investigated whether EF abilities can be changed through experience, with some notable successes. Most of this work has involved adult-led training or interventions, which allow children to practice EFs in an environment where adults provide some guidance. For example, children's working memory, or their ability to maintain and manipulate information across a delay, can be improved through short periods of targeted training (e.g., Holmes et al., 2009; Bergman Nutley et al., 2011). During such training, children are presented with sequences of spoken or visual stimuli. After a brief pause, the child is instructed to reproduce the sequence either in forward order (requiring maintenance of information, but no manipulation) or in reverse order (requiring maintenance and manipulation). After training, children often show better performance in similar tasks assessing the same skills (e.g., Holmes et al., 2009, 2010; Thorell et al., 2009; Bergman Nutley et al., 2011; reviewed in Diamond and Lee, 2011; Shipstead et al., 2012). In addition, children's cognitive flexibility, or ability to change tasks or strategies in response to new environmental demands, can be improved via interventions implemented in preschool curricula (Lillard and Else-quest, 2006; Diamond et al., 2007; Bierman et al., 2008; Röthlisberger et al., 2012). These curricula have ranged from partial-day, small-group sessions where children play games developed to exercise EFs (Röthlisberger et al., 2012), to comprehensive, full-day implementations, such as those found in Tools of the Mind (Diamond et al., 2007) and Montessori (Lillard and Else-quest, 2006) classrooms, where teachers are trained to scaffold developing EFs throughout the day. Relative to children in business-as-usual classrooms, children enrolled in such curricula have subsequently shown better performance in tasks where they must flexibly shift from one rule (e.g., sorting cards by their shape) to another (e.g., switching to sorting the cards by color).

Altering children's experiences with such training and interventions has thus led to improvements in children's externally-driven EF, where they are instructed on what to do (e.g., sort cards according to shape; remember a sequence of digits), and when (e.g., now switch and sort according to color; now recall the digits in reverse order). In the real world, children who have developed externally-driven EF might behave in a goal-directed way when given reminders. For example, a child might successfully put on a coat in the morning after a reminder from a caregiver. However, it is less clear how children's experiences relate to their development of more self-directed executive functioning, where they must determine on their own what goal-directed actions to carry out and when. A self-directed child, for example, might put a coat on just before going outside without being told what to do.

The development of self-directed EF is a critical part of growing up. Self-directed EFs develop later than externally-driven forms of executive control (Welsh et al., 1991; Jacques and Zelazo, 2001; Smidts et al., 2004; Snyder and Munakata, 2010; Chevalier et al., 2011), and prove to be more cognitively demanding, even in adults (e.g., Bryck and Mayr, 2005; Forstmann et al., 2005; Lie et al., 2006). Tasks assessing self-directed control typically provide an overall goal, but challenge participants to generate their own rules for how and when to employ EFs to achieve that goal. For example, in the verbal fluency task, which is a frequently-used and longstanding measure of EF (e.g., Troyer et al., 1997, 1998; Henry and Crawford, 2004; Sauzéon et al., 2004; Costafreda et al., 2006; Birn et al., 2010; Raboutet et al., 2010; Unsworth et al., 2010), participants are given a category (e.g., foods), and asked to produce as many words falling within that category as possible across a 1-min interval. To produce many items, participants may cluster responses (by grouping words that fall within the same semantic subcategory) and switch between subcategories when available exemplars are in short supply (e.g., Troyer et al., 1997, 1998; Abwender et al., 2001; Koren et al., 2005). Individuals must endogenously detect the need to switch (e.g., when they cannot think of more breakfast foods) and select what to switch to (e.g., desserts, vegetables, or fruits). Each process critically relies on generation of internal cues and becomes less executively demanding when external cues are instead provided (Randolph et al., 1993; Tremblay and Gracco, 2006; Snyder and Munakata, 2010). Consistent with this analysis of the self-directed nature of this task, switching among subcategories has been well-validated as the most executively-demanding component of verbal fluency tasks: switching (as opposed to naming items within clusters) activates prefrontal cortex (e.g., Hirshorn and Thompson-Schill, 2006), is impaired by prefrontal lesions (e.g., Troyer et al., 1998), and has the most protracted developmental course, with performance continuing to increase through adolescence (e.g., Kave et al., 2008). Young children often fail to switch from one subcategory to another, and instead perseverate on an initial subcategory (e.g., naming five different breakfast foods, and then indicating to the experimenter that they are finished). However, like patients with frontal lobe dysfunction, who benefit from semantic cueing during verbal fluency (Randolph et al., 1993; Drane et al., 2006; Iudicello et al., 2012), children can improve on the task when demands on self-directed EF are reduced by providing example subcategories prior to the task (Snyder and Munakata, 2010). This body of literature highlights the role of self-directed EF in switching among subcategories in the verbal fluency task.

We predicted that children's self-directed EFs might benefit from participation in less structured activities, where children, rather than adults, choose what they will do and when. Such experiences could support the practice of self-directed executive functioning, and lead to benefits. For example, children may practice engaging self-directed forms of EF by establishing goals and carrying them out across an afternoon (“first I'll read this book, then I'll make a drawing about the book, then I'll show everyone my drawing”) or during a visit to a museum (“first I want to see the dinosaur exhibit, and then I want to learn about rocks”). These types of self-directed choice and planning are central to the Tools of the Mind and Montessori classrooms, although the exact form they take and the types of activities emphasized differ across these programs (Montessori, 1976; Bodrova, 2003; Bodrova and Leong, 2007).

For example, extended, social pretend play figures centrally in the Tools of the Mind program. This program is based on the work of Vygotsky (Bodrova and Leong, 2007), who theorized that imaginative play supports the development of self-directed EF, in children's transitions from other-regulated to self-regulated cognitive processes (Vygotsky, 1967). During pretend play, children may practice engaging self-directed forms of EF by developing and maintaining their own goals to guide their behavior, even in the presence of conflicting environmental signals: a child who uses a wooden spoon as a wand maintains a pretend use while inhibiting a typical use (stirring a pot). Children's pretend play, as assessed during laboratory tasks with an adult experimenter, does predict their externally-driven EFs (Albertson and Shore, 2009; Kelly et al., 2011; Carlson et al., 2014); however, this relationship has been observed less reliably when pretend play is assessed during naturalistic play (e.g., Elias and Berk, 2002; Kelly et al., 2011; cf. Harris and Berk, as discussed in Lillard et al., 2013).

While preschool programs such as Tools of the Mind and Montessori implement the types of activities that we predict will benefit self-directed EFs, and such programs improve children's externally-driven EFs as discussed above, little work has investigated the relationship between such activities and the development of self-directed EFs. One study found that 12-year-old Montessori students were rated more highly on a measure of creativity than non-Montessori students, when writing answers to complete the prompt, “__ had the best/worst day at school” (Lillard and Else-quest, 2006). While such findings are suggestive because open-ended writing assignments have the potential to tap self-directed EFs, the prompt completion task is not an established measure of self-directed EFs, and there is some debate about the extent to which creativity reflects EF (e.g., Groborz and Nęcka, 2003; Chrysikou and Thompson-Schill, 2011; Ellamil et al., 2012; Jarosz et al., 2012). In addition, improved performance on this task may also reflect other benefits from such programs to language or writing skills; additional benefits were in fact observed on this task in the Montessori students' sentence sophistication. Moreover, it is unclear whether a broader range of less-structured activities outside of formal schooling yield EF benefits. Investigating this question is important, given that effects observed inside formal settings with trained adults may not generalize to other settings (as in the case of the pretend play effects discussed above), and given that not all families have access to the school settings where effects have been observed.

As a first step in examining the question of how children's experiences outside of formal schooling relate to EFs, we conducted a naturalistic, correlational study, in which we measured the time that 6-year-old children spent in their daily lives in structured and less-structured activities and tested whether it predicted performance in the lab on well-established executive function tasks, both externally-driven and self-directed. At this age, children spend some time in both structured and less-structured activities (e.g., Meeks and Mauldin, 1990; Hofferth and Sandberg, 2001a) and show some ability in self-directed control tasks, without showing high levels of proficiency (e.g., Welsh et al., 1991; Brocki and Bohlin, 2004; Kave et al., 2008; Snyder and Munakata, 2010, 2013).

To classify structured and less-structured activities, we relied on studies of child leisure time use (e.g., Meeks and Mauldin, 1990; Larson and Verma, 1999; Hofferth and Sandberg, 2001b; Fletcher et al., 2003; Osgood et al., 2005), which have attempted to discriminate between activities constituting structured, or constructive leisure, and “unstructured” leisure activities. “Unstructured” activities in this literature might be better thought of as “less-structured” activities, given that they can include some adult structuring, so we use the latter terminology throughout this paper. Most leisure time studies have identified structured leisure activities as those that are “supervised to some degree by a conventional adult, are highly structured, and provide [children] with a clear set of conventional activities in which to engage” (Agnew and Petersen, 1989, p. 335). Such activities “are… organized by adults around specific social or behavioral goals” (Fletcher et al., 2003, p. 641). Thus, structured time in the present study was defined to include any time outside of formal schooling^1^ spent in activities organized and supervised by adults (e.g., piano lessons, organized soccer practice, community service, homework). Less-structured activities have been described more loosely, and generally include voluntary leisure activities where adults provide fewer guidelines or direct instructions, such as activities that are “spontaneous, [taking] place without formal rules or direction from adult leaders, and [featuring] few goals related to skill development” (Mahoney and Stattin, 2000, p. 116). Our coding scheme follows existing coding schemes documented in Meeks and Mauldin (1990) and Hofferth and Sandberg (2001b). In cases where these coding schemes differed, we reviewed the literature to ensure that our coding was in accordance with the majority of other time use studies^2^. In the present study, less-structured activities included activities such as free play, family and social events, reading, drawing, and media time. While these classifications are imperfect (e.g., they do not capture the degree of structure within and across classifications—an issue we return to in the Discussion), they allow us to build on the existing literature, and serve as an important starting point for testing our predictions; further analyses allow us to test the importance of particular activities within these classifications.

We hypothesized that the amount of time children spent in less-structured activities would predict their self-directed EF, over and above any differences attributable to age, general vocabulary knowledge, and household income. We expected these effects to be specific, such that less-structured activities would not predict externally-driven EF and structured activities would not predict self-directed EF.

## Methods

### Participants

Seventy children participated in the study [*M*_age_ = 6.58 years; range = (6.01–7.00 years); males = 37]. All participants were recruited from a database of families who had volunteered to participate in research. During subject recruitment, parents were informed that they would be asked to document child activities during the week prior to the study visit. Three participants were excluded from analyses because detailed information on their weekly activities was unavailable, either because parents did not wish to provide this information (2), or because data were lost due to a technical error at the time of parent submission (1). Of the remaining participants, one child did not complete the Flanker task, one child did not complete the digit span task, and two children did not complete the verbal fluency task; each of these children was excluded from the analysis of only that task. All other participants completed all study tasks. Prior to their participation, parents gave informed consent, and children gave verbal assent. Children received small gifts (e.g., gliders, balls) throughout the project for their participation, and parents received $5 as compensation for travel.

### Design and procedure

Children were individually tested in a single session lasting approximately 1.5 h, with breaks given as needed. All children completed tasks in the same order: AX-CPT, Flanker, forward digit span (for other purposes, not discussed further in this report^3^), verbal fluency, and the Expressive Vocabulary Test. During the child tasks, parents provided demographic information and completed surveys of children's daily, annual, and typical schedules, as well as an exploratory “helicopter parenting” scale (not discussed further in this report; from Obradovic, pers. commun., October 26, 2011).

#### Parent questionnaires

***Parent survey of child time use***. Parents reported all child activities during the week prior to the laboratory test session using a computer-based survey. At the time that the study visit was scheduled, parents were informed that they would complete a detailed child activity survey during their visit, and were encouraged to take notes on their child's activities throughout the week. Parents were allowed to consult notes as they completed the survey. The survey was formatted as a 36 × 7 grid, such that each cell represented a 30-min time interval during the prior week (intervals occurring between midnight and 5:30 am were excluded to reduce burden). In each cell, parents wrote short, open-ended description of their child's activities, excluding times where children were sleeping or in school (parents indicated sleep and school schedules in a separate section of the survey). Before completing the survey, parents were asked to indicate the extent to which their family's activities over the prior week reflected typical patterns of time use. Parents rated their level of agreement with the prompt, “Was your family's schedule last week unusual or atypical?” via a 7-point scale anchored by “Strongly agree” and “Strongly disagree.” Parents were then given verbal and written instructions, as follows:

“Be as specific as possible for every activity you report. For example, for time spent in the car during a commute, rather than writing, “Drove from ___ to ___,” you could write, “Watched a DVD with his sister in the car while driving to the city for a research appointment.”

Indicate who your child was interacting with during a given activity. For example, if your child had free time to play outside between dinner and bedtime, rather than writing “Free time outside,” you could write, “Played tag outside with older sister and friends from the neighborhood.” Or, if your child reads before bedtime, rather than writing, “Reading time,” you could write, “Read aloud to mom before bed.”

Indicate simultaneous activities. For example, if your child ate a snack after school or camp while he/she had some down time, rather than writing “Snack time,” you could write, “Ate a snack while coloring.”

As parents completed the survey, experimenters periodically reviewed responses and asked that parents modify entries that were difficult to interpret or insufficiently detailed. Experimenters were also available during breaks between tasks to respond to parent questions about specific responses.

Child activity data were coded by three independent raters who were blind to data on all other tasks during each stage of the coding process. Coders assigned a numeric code to each cell-based survey entry using an activity classification scheme (Table 1). To ensure consistency across raters and reduce procedural drift, all raters independently classified each cell for the first 35 participants. Coders then met to discuss major discrepancies and to generate additional generalizable rules. Coders categorized responses from the final 32 participants using these agreed-upon criteria. The final 32 subjects were used to establish inter-rater reliability; reliabilities among pairs of coders ranged from 0.96 to 0.97, with coders agreeing on 7942 to 8021 cells out of 8288 total (i.e., 2 cells per hour × 18.5 h/day × 7 days a week × 32 participants). Excluding sleep and school cells (where there were no discrepancies between coders), reliabilities among pairs of coders were also high, ranging from 0.93 to 0.95. The three coders met to discuss discrepancies and generate a final, coded data set for each participant.

**Table 1:** **Classification of child time use (structured, less-structured, and other activities)**.

**STRUCTURED ACTIVITIES**
Physical lessons (e.g., soccer practice, karate)
Non-physical lessons (e.g., piano lessons, art class)
Tutoring
Homework and study
Chores
Religious activities
Other formal organizational meetings and activities (e.g., community service)
**LESS-STRUCTURED ACTIVITIES**
Unguided, child-initiated practice (e.g., playing piano or singing outside of scheduled practice times; shooting goals outside of soccer practice)
Free play alone
Free play with others
Social outings
Visits to family and friends
Parties
Camping
Picnics
Other group activities (e.g., walks, bike rides, skiing, swimming, bowling, golf)
Enrichment activities
Sightseeing
Aquarium and zoo visits
Museums
Miscellaneous educational events (e.g., science fair)
Other entertainment (e.g., live sporting events, performances, movies)
Reading
Media and screen time (e.g., TV, internet, video games)
**OTHER ACTIVITIES**
Sleeping
Meals/eating
School
Care by others
Personal care and hygiene
Child appointments
Commuting and travel time
Unknown/Unreported

*All entries that parents provided in the child time use survey were classified into these categories, following existing coding schemes (Meeks and Mauldin, 1990; Eccles and Barber, 1999; Mahoney and Stattin, 2000; Hofferth and Sandberg, 2001b; Fletcher et al., 2003; Osgood et al., 2005)*.

After the raters generated the final set of activity codes, each activity was further classified as either “Structured” or “Less-Structured” based on the coding scheme outlined in Table 1, following existing coding schemes (Meeks and Mauldin, 1990; Eccles and Barber, 1999; Mahoney and Stattin, 2000; Hofferth and Sandberg, 2001b; Fletcher et al., 2003; Osgood et al., 2005). All child-initiated activities (play, spontaneous practice, reading, watching television) and outings and events (museum or library visits, sporting events) were coded as “Less-Structured.” Adult-led lessons and practices, homework and studying, religious activities, and organization meetings (e.g., community service) were coded as “Structured.”

***Parent survey of typical child time spent in less-structured activities***. In a separate survey, parents were asked to indicate how often their children engaged in typical play activities by using a 7-point scale (“Never,” “Less than once a month,” Once a month,” “2–3 times a month,” “Once a week,” “2–3 times a week,” “Daily”) to rate the following items: “Surf the internet,” “Watch television, videos/DVD, or online media,” “Play video games (non-instructional),” “Play interactive instructional or learning games,” “Play with toys alone,” “Play with toys with friends/siblings,” “Play physical games with friends/siblings,” “Play physical games alone,” “Play non-physical games alone,” “Play card or board games with family,” “Read,” “Help with housework or cooking,” “Play musical instrument”, “Listen to music.” Scores on each item (where 1 = “Never” and 7 = “Daily”) were summed to produce a typical less-structured activity score.

***Parent survey of seasonal child activities***. In a separate survey, parents were asked to indicate the number of hours their child spent in structured lessons during the past year. Parents responded to 18 common structured lessons (basketball, baseball, tennis, hockey, soccer, football, golf, swimming, dance, gymnastics, martial arts, skiing/snowboarding, ice skating, music, art, theater, and tutoring) and were asked to write in any structured lessons that did not fall into these categories (most commonly, religious activities, and organizational meetings). To reduce burden, parents provided seasonal time estimations for each activity (e.g., the typical hours per week a child spent participating in music lessons over the prior fall). Data were reviewed for accuracy to ensure that parent-reported structured activities adhered to the same coding guidelines used to evaluate the Parent Survey of Weekly Activities. Cumulative hours spent in structured activities across the year were summed to produce an annual structured hours score.

***Household income***. Parents reported annual household income via an interval scale (median bracket: $100,000—$124,999; range: < $25,000 to > $150,000 USD). Fourteen parents chose not to disclose income information.

#### Child endogenous executive function measure

***Verbal fluency***. In the verbal fluency task, children were asked to generate words in response to a categorical prompt. The task was presented as a game to make it more engaging for children (as in Snyder and Munakata, 2013). Children were told, “We're going to play a game where we think of lots and lots of words. I bet you're really good at thinking of words, aren't you? I'll tell you what kinds of words to think of, and every time you tell me one, I'll put a pom-pom in your cup. Let's see how many pom-poms you can get before all the sand is gone (experimenter pointed to a 1-min sand timer children could use to estimate how much time was left). I'll bet you can get a lot! And when we are all done thinking of words, you can trade the pom-poms for a prize.” Before each category, the experimenter said, “This time I want you to tell me as many [category name] as you can think of. Can you think of lots and lots of [category name]? Ready, go!” The experimenter placed a pom-pom in a clear plastic cup in front of the child for each new exemplar. If children paused for 10 s or longer between items, they were encouraged to continue (“Good job, can you tell me some more [category name]?”). In the rare instance where a child stated that she/he had named all words, the experimenter double-checked with the child (e.g., “Are you sure? What other [category name] can you think of?”) and waited with the child until the end of the block. Children completed three blocks using this procedure, each of 1-min duration: a practice block (with the prompt “household items”), and two test blocks (with the prompts “animals” and “foods,” which were counterbalanced across participants).

Verbal fluency data were transcribed from audio recordings, and coded by the experimenter and two independent raters blind to data on all other tasks. Coders identified clusters of items that were semantically related (e.g., “cookies, pie, cake” when producing foods). Switches between clusters of related items were identified and summed to generate cumulative switch scores. Switch scores were weighted by cluster size (as in Snyder and Munakata, 2010, 2013), such that 1 point was awarded for a switch after a cluster of 2 related items, 2 points for a switch after 3 related items, 3 points for switch after 4 related items, and so on. Weighted switch scores were used because they reflect increasing confidence as cluster size increases that children are indeed clustering and switching. Unweighted scoring systems (e.g., Troyer et al., 1997), which count every transition between subcategories equally (including between single, unclustered items), have been criticized for confounding switching with a failure to cluster (e.g., Abwender et al., 2001). Inter-rater reliabilities were high between all pairs (>85%). To generate cumulative switch scores for each participant, weighted switch scores were averaged across coders within each prompt, and then summed.

#### Child externally-driven executive function measures

***Flanker***. Children completed a computerized flanker task (Eriksen and Schultz, 1979) assessing their ability to resolve conflicting visual information by appropriately responding to a central stimulus while ignoring flanking stimuli. The Flanker task is a commonly-used measure of externally-directed EF in 6-year-olds (e.g., Ridderinkhof and van der Molen, 1995; Rueda et al., 2004, 2005; McDermott et al., 2007; Röthlisberger et al., 2012) and has been shown to be sensitive to some interventions targeting EF in this age group (Fisher et al., 2011; Röthlisberger et al., 2012). During the task, children were instructed to indicate the orientation (left or right pointing) of a centrally-presented target stimulus, via a corresponding button press. In congruent trials, the target stimulus (the center fish) was surrounded by fish with the same orientation. In incongruent trials, the target image was surrounded by fish with an opposite orientation. In neutral trials, only the target image was presented and was not surrounded by any fish. Following a 10-trial practice block (4 congruent, 4 incongruent, 2 neutral), children completed three 32-trial blocks of the task: two incongruent blocks (for each block, incongruent trial *N* = 16; neutral trial *N* = 16), separated by one congruent block (congruent trial *N* = 16; neutral trial *N* = 16). Trials were presented in random order within blocks.

Reaction times were used to assess children's ability to resolve interference among conflicting stimuli, as in past work with this age group (e.g., Rueda et al., 2005; McDermott et al., 2007; Röthlisberger et al., 2012). Incongruent trials require children to attend to only the target middle fish and to ignore the surrounding fish. Therefore, the flanker task can be used to assess children's ability to filter out irrelevant information. Larger interference costs (i.e., the difference between average response time on incongruent trials and average response time on neutral trials) reflect greater difficulty filtering irrelevant information. To assess filtering ability, we first calculated participant mean response times for each trial type (neutral, incongruent/congruent) within each block across trimmed, correct trials (trials <100 and >3000 ms were excluded, as well as any trials three standard deviations outside that participant's mean for that trial type and block). To generate robust estimates of possible interference effects (as suggested by Lavie, 1995, and implemented in D'Ostilio and Garraux, 2012), incongruent/congruent trial mean RTs were contrasted with neutral trial mean RTs from the same block, yielding one congruent-neutral contrast and two incongruent-neutral contrasts within each participant. Flanker conflict scores were generated by subtracting the congruent contrast from each incongruent contrast (yielding two conflict scores, one arising from each incongruent block). These conflict scores were averaged to generate a summary flanker conflict score.

***AX-CPT***. Children completed the AX Continuous Performance Task (AX-CPT), which provides a measure of proactive control, or the tendency to maintain goal-relevant information until it is needed (Braver et al., 2007). All procedures and analyses were conducted as in Chatham et al. (2009). In this touchscreen-based, child-friendly version, children are allowed to prepare for future circumstances (the appearance of either “X” or “Y” image probes) based on previous experiences (the appearance of “A” or “B” image cues).

Children were instructed to respond with a target response whenever the “A” context cue was followed by an “X” probe. Children were instructed to provide a non-target response to all other cue-probe sequences (A – Y; B – X; B – Y). To improve child engagement during the task, popular cartoon characters were used as image stimuli, and the instructions took the form of character preferences. For example, children were told, “Spongebob likes watermelon, so press the happy face when you see Spongebob and then the watermelon,” and, “Blue doesn't like the slinky, so press the sad face when you see Blue and then the slinky.”

After the experimenter explained the task rules, children completed a “verification” phase to ensure that they understood the instructions and were capable of following rules. During this phase, each cue–probe pair was presented sequentially, and participants were asked to indicate the correct response for each pair. If subjects responded incorrectly to a cue-probe pair, the experimenter repeated the relevant rule (“Remember, when you see [A, B] and then you see [X, Y], tap this button [appropriate button blinks] as quickly as you can!”) and subjects were allowed to try again. Participants then completed 7 practice trials. Cues were presented for 500 ms, followed by a 120 ms delay period, and a subsequent 6 s probe, as in test trials. Test trials were presented in four 30-trial blocks, where 70% of trials were target (A – X) trials, and 30% were non-target trials (A – Y; B – X; B – Y, appearing in equal proportion).

Proactive children show a characteristic behavioral profile that can be used to generate an RT-based measure of proactive control. Children who engage proactive control generate fast RTs in BX and BY trials, since maintenance of the “B” cue supports a non-target response to the subsequent “X” probe, and slower RTs on AY trials, since active maintenance of the “A” cue leads to anticipation of an “X” probe (due to the expectancy generated by asymmetric trial type frequencies). Proactive control was thus calculated using the median of trimmed RTs on correct AY and BX trials, which were entered into the formula (AY – BX)/(AY + BX). All responses made <200 ms after the presentation of the probe were removed from the analysis, resulting in the exclusion of <1% of all trials.

***Expressive vocabulary test***. The EVT (Pearson Assessments, Bloomington, MN) is a standardized, nationally normed, expressive vocabulary test, which we used (as in Snyder and Munakata, 2010) to control for differences in vocabulary that might have influenced verbal fluency performance (i.e., a child with a robust vocabulary might be capable of generating larger clusters than a child with a limited vocabulary, independent of either child's switching ability). On each trial of the EVT, children are shown a colored picture and are asked to name it or provide a synonym (e.g., “Can you tell me another word for father?”). Testing continues until children incorrectly answer five items in a row, and raw scores are then converted into a standardized score based on age.

## Results

### Preliminary results and analysis approach

**Figure 1:**
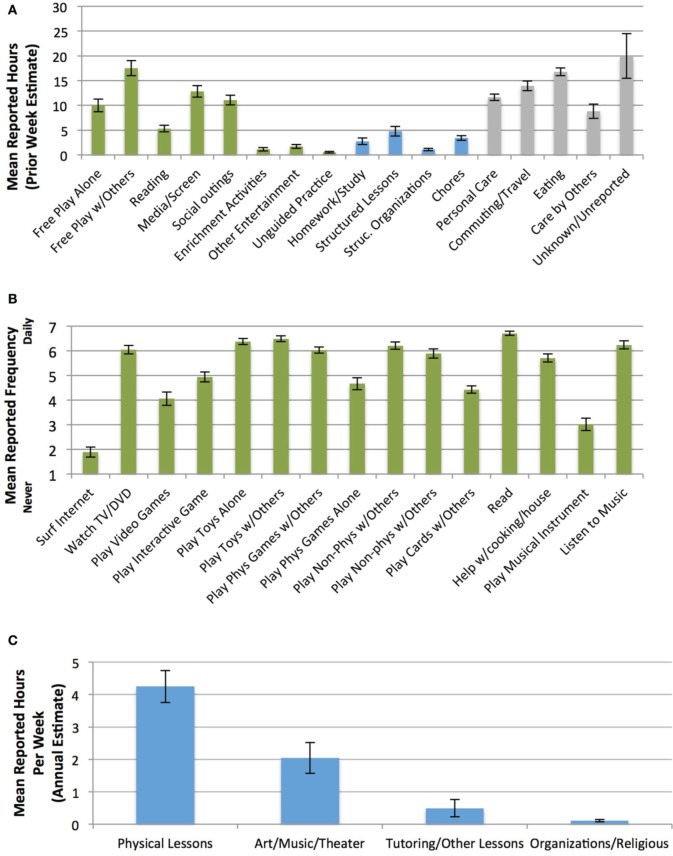
**Parent-reported child time use. (A)** Activities in week prior to laboratory visit (green, less-structured; blue, structured; gray, other). **(B)** Typical less-structured activities (1, Never; 2, Less than once a month; 3, Once a month; 4, 2–3 times a month; 5, Once a week; 6, 2–3 times a week; 7, Daily). **(C)** Typical structured activities during a typical week (averaged across 4 seasons). Prior-week and typical measures of parent-reported child time use were correlated and combined into z-scored composite estimates of structured and less-structured time. For all figures, error bars indicate standard error of the mean.

Weekly and annual/typical estimates of how children spent their time (Figures 1A–C) were marginally correlated, for both structured activities (*r* = 0.24; *p* < 0.06) and less-structured activities (*r* = 0.23; *p* < 0.071). We thus generated composite scores across weekly and annual/typical estimates to provide a more accurate and reliable measure of children's time. Each composite measure (for structured time, and separately for less-structured time) was formed by summing z-scored time in prior-week activities with z-scored ratings from the parent survey of annual/typical child activities, within each participant.

All analyses were conducted using standard linear regression. We included age, gender, and family income as factors in all models, given that they or related factors are often predictive of children's EF: age (e.g., Welsh et al., 1991; Huizinga et al., 2006), gender (e.g., Blair et al., 2005; Diamond et al., 2007), family income Hughes et al., 2009; as a component of SES: (Farah et al., 2006; Noble et al., 2005, 2007; Raver et al., 2013). Child vocabulary, as indexed by EVT performance, was included as a covariate in all tests of verbal fluency performance. Descriptive statistics for executive function, vocabulary, and time use measures are given in Table 2. Individual EF measures were not correlated, before or after controlling for age (*p*'s > 0.4). For all analyses, outlying observations were identified (Cook's D > 3 standard deviations above the mean) and removed. This resulted in the exclusion of no more than four cases from any analysis.

**Table 2:** **Descriptive statistics for executive function, vocabulary, and time use measures (*N*'s = 65–67)**.

**Measure**	**Mean (*SD*)**
**SELF-DIRECTED EF**	
Verbal fluency combined switch score	10.13 (4.1)
**EXTERNALLY-DRIVEN EF**	
AX-CPT proactive control score	0.094 (0.12)
Flanker conflict score	164.5 (168.7)
Vocabulary: EVT standardized score	112.9 (9.4)
**PRIOR WEEK CHILD TIME USE**	
Structured hours	6.03 (5.9)
Less-structured hours	32.2 (14.2)
Typical child less-structured activities (combined score)	78.5 (8.8)
Seasonal child structured activities (annual hours)	91.5 (89.0)

### Child time use and self-directed EF

#### Less-structured time

**Figure 2:**
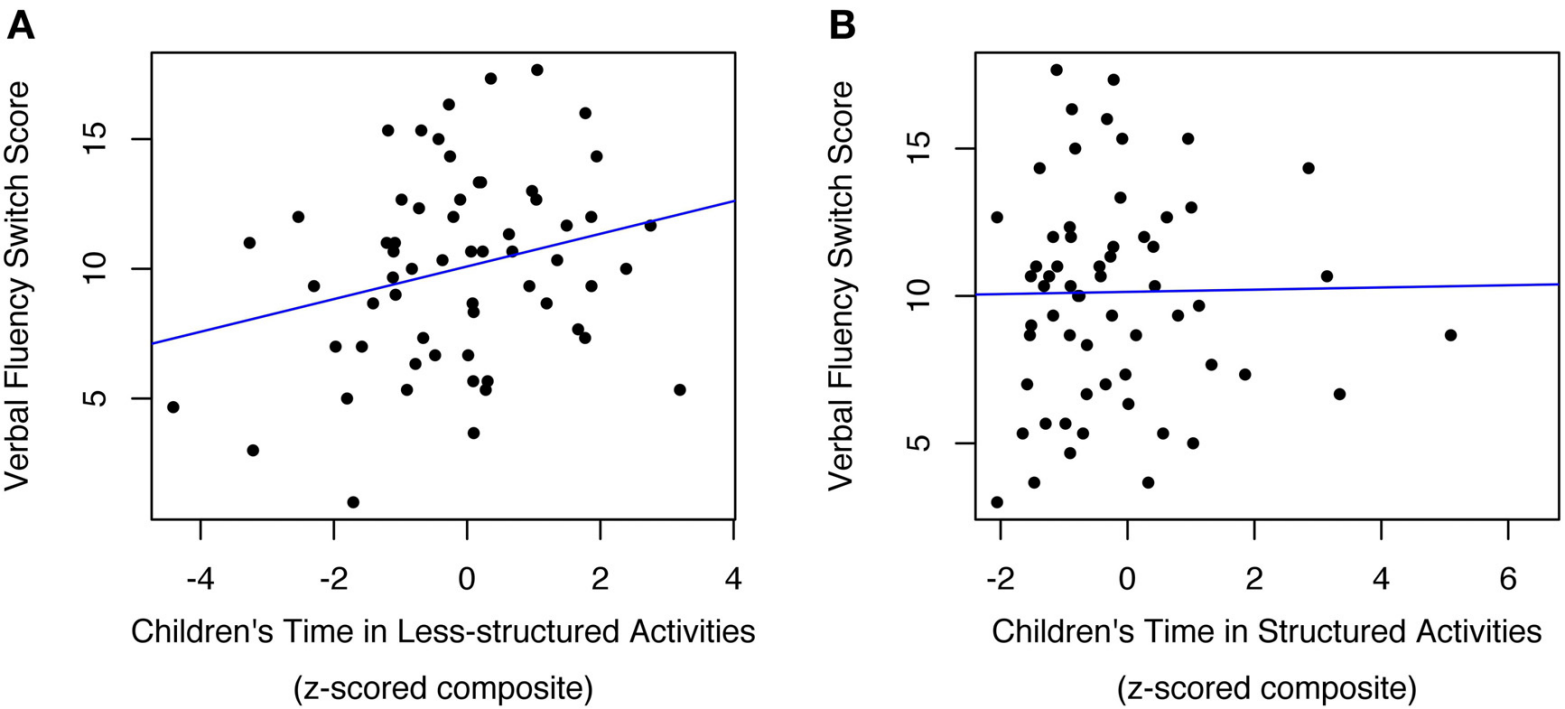
**Children's self-directed EF (as measured in Verbal Fluency) was predicted by more time spent in less-structured activities (A), and marginally predicted by less time spent in structured activities, although this relationship is not apparent because the figure does not capture how the effects of age, income, gender, and EVT were controlled for in all analyses (B)**. Outlying observations have been excluded [*N* = 3 in **(A)**; *N* = 2 in **(B)]**.

As predicted, children who spent more time in less-structured activities demonstrated better self-directed EF, as indexed by verbal fluency performance [η^2^_*p*_ = 0.07; *F*_(1, 44)_ = 4.46; *p* < 0.05; Figure 2A; Table 3]. In addition, older children and children with higher vocabulary scores demonstrated better verbal fluency performance [Age: η^2^_*p*_ = 0.11; *F*_(1, 44)_ = 7.45; *p* < 0.01; EVT: η^2^_*p*_ = 0.10; *F*_(1, 44)_ = 6.30; *p* < 0.02]. In subsequent tests for interactions, we found an unexpected interaction between less-structured time and age [Less-structured time × Age: η^2^_*p*_ = 0.08; *F*_(1, 43)_ = 5.48; *p* < 0.03]. *Post-hoc* tests indicated that additional time in less-structured activities predicted better self-directed control in most but not all children; specifically, this finding held in both the youngest sample quartile [*M*_age_ = 6.38 years, Less-structured time: η^2^_*p*_ = 0.07; *F*_(1, 43)_ = 10.37; *p* < 0.003] and at the median [*M*_age_ = 6.65 years, Less-structured time: η^2^_*p*_ = 0.07; *F*_(1, 43)_ = 6.81; *p* < 0.02], but not in the oldest quartile (*M*_age_ = 6.86 years; *p* > 0.8). When the interaction between less-structured time and age was included in the model, children from higher-income households demonstrated marginally better verbal fluency performance {Income: η^2^_*p*_ = 0.05; [*F*_(1, 43)_ = 3.36; *p* < 0.08]}. Age, vocabulary, and time in less-structured activities also continued to predict self-directed EF [Age: η^2^_*p*_ = 0.12; *F*_(1, 43)_ = 5.76; *p* < 0.03; Vocabulary: η^2^_*p*_ = 0.07; *F*_(1, 43)_ = 4.80; *p* < 0.04; Less-structured time: η^2^_*p*_ = 0.07; *F*_(1, 43)_ = 6.81; *p* < 0.02].

**Table 3:** **Effects of age, gender, income, vocabulary and time use on child verbal fluency performance**.

	**Age, income, and gender**			**EVT, age, income, and gender**			**Less-structured time + age, income, gender, and EVT**		
**Variable**	β	***t^b^***	***P***	β	***t^b^***	***P***	β	***t^b^***	***P***
(Intercept)	9.99	22.48	<0.001^***^	9.95	22.13	<0.001^***^	9.94	22.67	<0.001^***^
Age (days)	0.008	1.79	<0.09	0.013	2.95	<0.01^**^	0.011	2.73	<0.01^**^
Gender (1 = female; −1 = male)	0.008	0.02	>0.9	−0.194	−0.42	>0.6	−0.392	−0.82	>0.4
Household income	0.743	3.17	<0.01^**^	0.301	1.13	>0.2	0.372	1.48	>0.1
Vocabulary (EVT)	–	–	–	0.177	2.78	<0.01^**^	0.142	2.51	<0.05^*^
Less-structured time	–	–	–	–	–	–	0.713	2.11	<0.05^*^
Less-structured time x age	–	–	–	–	–	–	–	–	–
Structured time	–	–	–	–	–	–	–	–	–
Model *F*-value	4.05			4.88			4.56		
Model Adjusted *R*^2^	0.16			0.24			0.27		
	**Less-structured time × age + income, gender, and EVT**			**Structured time + age, income, gender, and EVT**					
**Variable**	β	***t^b^***	***P***	β	***t^b^***	***P***			
(Intercept)	10.09	23.86	<0.001^***^	9.73	21.97	<0.001^***^			
Age (days)	0.010	2.40	<0.05^*^	0.012	2.90	<0.01^*^			
Gender (1 = female; −1 = male)	−0.375	−0.82	>0.4	−0.169	−0.36	>0.7			
Household income	0.442	1.83	<0.08	0.487	1.87	<0.07			
Vocabulary (EVT)	0.120	2.19	<0.05^*^	0.128	2.24	<0.05^*^			
Less-structured time	0.854	2.61	<0.05^*^	–	–	–			
Less-structured time x age	−0.008	−2.34	<0.05^*^	–	–	–			
Structured time	–	–	–	−0.596	−1.89	<0.07			
Model *F*-value	5.10			4.34					
Model Adjusted *R*^2^	0.33			0.26					

Age, income, EVT scores, and less-structured and structured time composite scores are mean-centered. For each model, observations where Cook's D > 3 standard deviations above the mean were identified and removed. N_Model1_ = 45; N_Models2–3_ = 44; N_Models4–5_ = 43;

* p < 0.05;

** p < 0.01;

*** *p < 0.001*.

***Exploratory analyses***. We next investigated whether specific kinds of less-structured activities were driving the observed relationship between less-structured time and self-directed control. Composite variables representing common less-structured activities were created by aggregating similar responses across prior-week and annual/typical measures^4^. This procedure yielded seven broad categories of less-structured activities: unguided practice; play alone; play with others; social events with family (including parties, camping, picnics, and other group outings, such as hiking, biking, and swimming^5^), enrichment events (visits to the museum, library, aquarium, or zoo; sightseeing; and miscellaneous educational events), other entertainment (movies, performances, and live sporting events); reading; and media and screen time. Enrichment activities [η^2^_*p*_ = 0.11; *F*_(1, 44)_ = 6.95; *p* < 0.02] and social events [η^2^_*p*_ = 0.10; *F*_(1, 43)_ = 7.26; *p* < 0.01] significantly predicted self-directed EF, and play with others was marginally predictive [η^2^_*p*_ = 0.05; *F*_(1, 44)_ = 3.42; *p* < 0.072]. Interactions with age were not significant in these models, and were therefore excluded (*p*'s > 0.2). No other classes of less-structured activities predicted verbal fluency performance.

We then considered whether the relationship between less-structured time and self-directed EF persisted when we excluded from our less-structured time composite measure, in sequential analyses:

1. media and screen time (which might reflect passive, rather than self-directed leisure activity);
2. activities within the less-structured time classification that may have included more structure than other such activities; and
3. enrichment activities that may have yielded benefits specific to verbal fluency performance (rather than self-directed control, *per se*).

When media and screen time were excluded, less-structured time continued to demonstrate a positive relationship with self-directed EF [η^2^_*p*_ = 0.06; *F*_(1, 41)_ = 5.23; *p* < 0.03]. This finding persisted when we also excluded less-structured activities that may have included more structure than other such activities (e.g., board games played with a group; rule-based physical games such as golf and bowling; movies and performances; reading with others^6^) [η^2^_*p*_ = 0.06; *F*_(1, 43)_ = 6.17; *p* < 0.02]. As a final step, we also excluded visits to museums, aquariums, and zoos, which may have benefitted organization of semantic clusters on the verbal fluency task (e.g., exposure to zoo animals may have helped to organize animal clusters, and thus yielded performance benefits). Using this fully-restricted measure of less-structured time, child time in less-structured activities continued to predict better self-directed EF [η^2^_*p*_ = 0.06; *F*_(1, 43)_ = 6.23; *p* < 0.02]. Interactions with age were significant and were included in each of these restricted analyses (all *p*'s < 0.05).

We also explored whether participation in types of less-structured activities changed with age, and whether such changing patterns of time use could speak to the diminished link between less-structured time and self-directed control in the oldest quartile of children in our sample. Media and screen time use was more prevalent in older children [η^2^_*p*_ = 0.05; *F*_(1, 61)_ = 5.15; *p* < 0.03]. Time spent in other categories of less-structured activities did not vary with age (*p*'s > 0.2).

#### Structured time

Additional time in structured activities predicted marginally worse self-directed control [η^2^_*p*_ = 0.06; *F*_(1, 43)_ = 3.57; *p* < 0.07; Figure 2B; Table 3]. Again, self-directed EF was predicted by age [η^2^_*p*_ = 0.13; *F*_(1, 43)_ = 4.43; *p* < 0.01], and vocabulary [η^2^_*p*_ = 0.08; *F*_(1, 43)_ = 5.02; *p* < 0.04], and marginally predicted by household income [η^2^_*p*_ = 0.05; *F*_(1, 43)_ = 3.50; *p* < 0.07]^7^.

***Exploratory analyses***. We next examined whether the relationship between structured time and self-directed EF persisted when we excluded religious services and household chores, where children may have been supervised less often by adults, relative to other structured activities. Time in structured activities continued to predict worse self-directed EF when religious services and chores were excluded from the composite structured time measure [η^2^_*p*_ = 0.06; *F*_(1, 43)_ = 4.28; *p* < 0.05].

### Child time use and externally-driven EF

**Figure 3:**
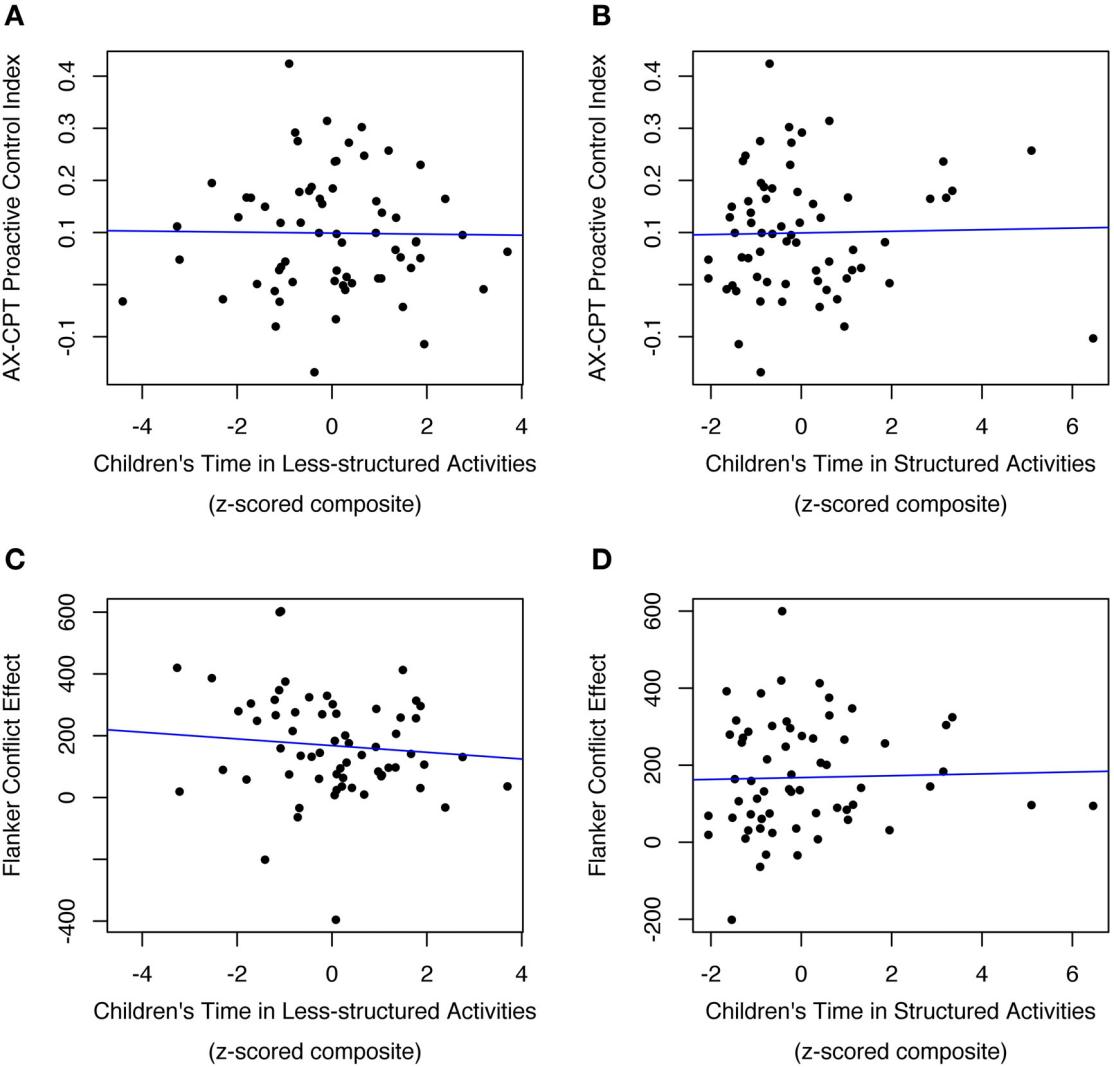
**Children's externally-driven EF (as measured in AX-CPT and Flanker) was not predicted by their time spent in either less-structured activities (A,C) or structured activities (B,D)**. Outlying observations have been excluded [*N* = 1 in **(A,B)**; *N* = 2 in **(C,D)**].

No measure of child time predicted any aspect of externally-driven EF (Figures 3A–D). Specifically, child time spent in less-structured activities did not relate to performance on either externally-driven EF measure (Flanker conflict score: *p* > 0.2; AX-CPT proactive control score: *p* > 0.8). Similarly, time in structured activities was unrelated to externally-driven EF (Flanker conflict score: *p* > 0.6; AX-CPT proactive control score: *p* > 0.3)^8^. Males demonstrated better Flanker conflict scores than females [η^2^_*p*_ = 0.10; *F*_(1, 46)_ = 4.64; *p* < 0.04]. No other variables predicted externally-directed EF^9^.

## Discussion

Our findings offer support for a relationship between the time children spend in less-structured and structured activities and the development of self-directed executive function. When considering our entire participant sample, children who spent more time in less-structured activities displayed better self-directed control, even after controlling for age, verbal ability, and household income. By contrast, children who spent more time in structured activities exhibited poorer self-directed EF, controlling for the same factors. The observed relationships between time use and EF ability were specific to self-directed EF, as neither structured nor less-structured time related to performance on externally-driven EF measures. These findings represent the first demonstration that time spent in a broad range of less-structured activities outside of formal schooling predicts goal-directed behaviors not explicitly specified by an adult, and that more time spent in structured activities predicts poorer such goal-directed behavior. Consistent with Vygotskian developmental theory and programs that build on that theory, such as Tools of the Mind, less-structured time may uniquely support the development of self-directed control by affording children with additional practice in carrying out goal-directed actions using internal cues and reminders. That is, less-structured activities may give children more self-directed opportunities. From this perspective, structured time could slow the development of self-directed control, since adults in such scenarios can provide external cues and reminders about what should happen, and when.

Surprisingly, the relationship between less-structured time and self-directed control changed with age in our participant sample, such that less-structured time predicted self-directed control in all but the oldest quartile of participants. This interaction between less-structured time and age was reliably observed across increasingly restrictive measures of less-structured time. One interpretation is that most but not all age groups within our sample spent their less-structured time in activities that encourage the development of self-directed control. Indeed, despite a relatively limited age range, our sample demonstrated differences in the content of less-structured time across 6–7 years of age, with older children spending more time engaged in media and screen activities. However, time spent in unguided practice, enrichment outings, and some forms of play was the main driver of the relationship between less-structured time and self-directed control in our data, and time spent in such activities did not change as a function of age. Another possibility is that children who have less developed self-directed control are more likely to benefit from less-structured time (in the same way that some interventions show the greatest benefits to children who show the worst initial performance, Connor et al., 2010; Diamond and Lee, 2011; cf. Bierman et al., 2008), such that the oldest and most advanced quartile of participants showed the least benefit.

While promising, it will be important for the present findings to be replicated and extended to address a number of limitations. For example, our sample came primarily from an affluent, suburban sample. This sample nonetheless included a broad enough range of incomes that income was predictive of self-directed EF, and the relationship between less-structured time and self-directed EF held even when controlling for income. However, less-structured time may be especially beneficial to children in safe, quiet, resource-rich environments, so it will be important to test whether it differentially relates to self-direction in more impoverished environments. In addition, although the current test of the relationship between less-structured time and self-directed EFs emerged from a targeted hypothesis, we conducted multiple post-hoc exploratory analyses to explore the relationship between specific activities and self-directed control, which are not ideal conditions for statistical inference.

Another limitation of the present study relates to our constructions of less-structured and structured time, which are imprecise, and most likely fail to capture important differences across activities. The broad, standardized definitions of structured and less-structured time adopted in this study (e.g., Meeks and Mauldin, 1990) ignore differences in the degree of independence that children experience within and across activities. In the present study, trips to museums, libraries, and sporting events are each classified as less-structured, but may vary in relative structure. That is, a typical library visit, where children may select their own sections to browse and books to check out, may involve much less structure (and more self-directed time) than a typical sporting event, where attention is largely directed toward the action on the field or court. Similarly, although any activity within the category of “media and screen time” counts as less-structured time, this category includes activities that range from passive movie-watching to self-directed internet searches to more structured video games. Even those activities that seem less-structured by definition, such as free play, can quickly become more structured when adults, older siblings, or peers impose additional rules or criteria. Indeed, many programmatic interventions have highlighted the importance of some structure to improve the quality of children's play and other learning experiences, and produce benefits (Schweinhart et al., 2005; Lillard and Else-quest, 2006; Diamond et al., 2007; Heckman et al., 2010; Lillard, 2012).

We note however, that even though our classification system based on the existing literature does not capture these variations in exactly how structured various activities are, our primary finding of the relationship between less-structured time and self-directed EF holds across analyses dropping potentially more difficult-to-interpret classifications (e.g., media and screen time, various games, movies and performances, and visits to museums, aquariums and zoos). To generate a more precise estimate of the amount of time children spend pursuing activities in a self-directed way, one would ideally assess child time directly, possibly by supplementing parent-reported child time use data with direct observation. One possibility along these lines could be to employ experience sampling techniques (Miller, 2012), where parents are frequently queried (via cell phone or another mobile device) throughout the day and asked to provide specific detail about their child's activities in the moment. Such methods would also minimize the need to rely on a parent's memory for their child's daily activities and experiences. We view our work as providing an important starting point for this kind of more time-intensive study of children's time outside of formal schooling and its relationship to their self-directed EF.

In addition, although we have identified links between child time use and self-directed EF, we are unable to draw firm conclusions about whether the observed relationships were driven by activities occurring in the week preceding the test session (as has been observed in other domains, e.g., Berns et al., 2013; Mackey et al., 2013), activities occurring over a longer period, or some combination. We used composite measures incorporating both recent and more distal/typical experiences, given that these measures were correlated and in an attempt to maximize the accuracy and reliability of parental estimates. We can test which one is more predictive of self-directed EF, recent or more distal/typical experiences, but it is difficult to make strong claims based on such analyses. For example, when examining less-structured activities and self-directed EF, we find that recent experiences predict self-directed EF [*F*_(1, 60)_ = 6.10; *p* < 0.02], but typical experiences do not (*p* > 0.6). This finding could reflect the greater importance of recent experiences, or it could reflect the greater precision of the time-diary measure, which indexes recent experiences but is also representative of more distal/typical experiences^10^. Similarly, when examining structured activities and self-directed EF, we find that neither recent nor annual experiences alone predict self-directed EF (*p*'s > 0.2). This finding could reflect the importance of the combination of recent and distal experiences, or simply the greater robustness of using a composite measure. Therefore, while we have posited that less-structured experiences allow children to practice self-directed, goal-oriented behavior, producing benefits over time, we cannot discount the possibility that observed linkages may have been driven by recent experiences which increased self-directed behavior. In either scenario, regular participation in less-structured activities would yield benefits.

Future investigations of the relationship between self-directed control and less-structured time would also benefit from the inclusion of additional measures of self-directed control, which more closely approximate real-world child behaviors. This process may benefit from the development and validation of new measures of self-directed control in children. Establishing effects using tasks tapping other forms of self-direction would also ensure generalizability. For instance, in the present study, time in less-structured activities such as family outings may have benefitted verbal fluency performance in a specific way, by fostering the development of more well-organized semantic networks, rather than by more generally improving children's abilities to generate their own rules for how and when to employ EFs to achieve their goals. This alternative account cannot explain the full pattern of results in the link between less-structured time and self-directed EF (e.g., the fact that this link persists when enrichment activities are excluded, and other less-structured categories such as unguided practice and play predict self-directed EF); however, a broader range of measures could provide a more robust and generalizable assessment of self-directed EF.

The findings of the current study are consistent with previous research in showing a link between children's experiences and EF (Lillard and Else-quest, 2006; Diamond et al., 2007; Bierman et al., 2008; Holmes et al., 2009; Bergman Nutley et al., 2011; Diamond, 2012; Röthlisberger et al., 2012; Zelazo and Lyons, 2012; Titz and Karbach, 2014). However, while the current study found specific effects of time use on self-directed but not externally-driven EF, previous research found effects of training and preschool interventions on externally-driven EF (e.g., see discussion in Diamond, 2012), but did not evaluate self-directed EF. There are several possible reasons for this discrepancy. First, previous training studies that have shown benefits for externally-driven EF have specifically trained children on aspects of externally-driven EF (e.g., working memory span tasks; e.g., Holmes et al., 2009; Bergman Nutley et al., 2011). Likewise, while preschool and other interventions include a wide variety of experiences, they likely include considerable practice with externally-driven EF. In contrast, we hypothesize that less-structured time primarily affords children practice with self-directed EF, and thus may not transfer to improving externally-driven EF. Second, it is possible that differences between the current versus previous studies could be accounted for by differences between the externally-driven EF tasks they employed. Many previous studies that have found effects of interventions on externally-driven EF used task-switching or working memory span tasks (e.g., Lillard and Else-quest, 2006; Diamond et al., 2007; Bierman et al., 2008; Holmes et al., 2009, 2010; Thorell et al., 2009; Bergman Nutley et al., 2011; Röthlisberger et al., 2012), whereas the current study used tasks assessing proactive control (AX-CPT) and conflict resolution (Flanker). It may be that specific aspects of externally-driven EF are more sensitive to children's experiences, or that specific tasks are more sensitive to individual differences in general due to better psychometric properties^11^. Future research using a more comprehensive battery of EF tasks could address these possibilities.

Another key difference between our study and such prior research is the correlational nature of our study, which supports at least two alternatives to the interpretation that how children spend their leisure time shapes their EF. First, children with better self-directed EFs may engage in (or be encouraged to engage in) less-structured activities more often. Likewise, children with poorer self-directed control may be more likely to engage in structured activities. Alternatively, the observed relationship between less-structured time and self-directed control may be driven by a third, unmeasured variable. Although we have attempted to control for some characteristics that might influence both time spent in less-structured activities and verbal fluency, such as household income, we have not controlled for other possibilities, such as parent EF and child's fluid intelligence (which we did not assess). However, we did control for child vocabulary (an index of crystallized intelligence), which may serve as a proxy for fluid intelligence in testing relationships with EF, given that EF fully mediates the correlation between crystallized and fluid intelligence in 7-years-old (Brydges et al., 2012)^12^. Moreover, such factors might be expected to predict both children's self-directed EF and their externally-driven EF (Ardila et al., 2000; Mahone et al., 2002; Kalkut et al., 2009), and so seem unlikely to explain why less-structured time predicts only the former. Similar issues have been raised in interpreting links observed between children's EF and pretend play: rather than reflecting a uniquely causal role for pretend play in EF, EF may instead play a causal role in supporting pretend play, or pretend play may be one of many activities promoting EF development in young children (Lillard et al., 2013).

An important direction for future work lies in establishing the directionality of relationships between child time use and self-directed EF, through experimental manipulation. Longitudinal studies could provide the first step toward establishing directionality. Specifically, if time spent in less-structured activities prospectively predicts change in self-directed EF, this would suggest that less-structured time may play a causal role in the development of self-directed EF. If, on the other hand, self-directed EF prospectively predicts changes in the amount of time children spend in less-structured activities, this would suggest that self-directed EF may play a causal role in children's time use (e.g., because parents might allow children with strong self-directed EF skills to play with less supervision). While such longitudinal studies could thus provide important information about temporal precedence, this information is not sufficient evidence of causality (e.g., additional unmeasured variables could actually be the causal factors). Thus, future research using experimental manipulations of time spent in less-structured activities is necessary to definitively test causality. One approach would be to attempt to randomly assign children to more structured or less structured environments, such as summer camps, where child activities could be carefully monitored via regular sampling of staff and/or on-site observation. Although this kind of work is ambitious, and poses challenges, it could be used to inform more targeted laboratory-based training studies.

Finally, we hope that future explorations of the relationship between child time use and developing self-directed EFs will inform a wider question: specifically, whether societal shifts in child time use over the past 50 years have influenced development. Hours formerly devoted to less-structured, social play have been replaced by media time (Vandewater et al., 2007; Bavelier et al., 2010; Hofferth, 2010; Johnson, 2010), and structured, adult-led activities (Hofferth and Sandberg, 2001a; Larson, 2001; Bianchi et al., 2006). Some commentators have warned that these changes have been to the detriment of children (e.g., Ginsburg, 2007; Milteer and Ginsburg, 2012). Others have argued that children benefit more from regular skill practice in structured settings (e.g., Chua, 2011; Ramdass and Zimmerman, 2011). Our findings indicate that during children's time outside of formal schooling, participation in less structured activities may benefit the development of self-directed EFs, while participation in structured activities may hinder the development of self-directed EFs. Thorough testing of this hypothesis remains an important direction for future work.

## Author contributions

Jane E. Barker, Andrei D. Semenov, and Yuko Munakata contributed to the development of the study hypothesis. All authors contributed to study design. Jane E. Barker performed the data analysis and drafted the manuscript with input from Yuko Munakata. Critical revisions were contributed by Hannah R. Snyder, Laura Michaelson, and Yuko Munakata. All authors discussed the results, implications, and literature, and approved the final version of the manuscript for submission.

### Conflict of interest statement

The authors declare that the research was conducted in the absence of any commercial or financial relationships that could be construed as a potential conflict of interest.

## Footnotes

^1^We did not classify time spent in school as “structured” because the degree of structure in school settings can vary a great deal, and parent reports are likely to be inaccurate (since parents often do not have direct knowledge of child activities during schooling hours). Our definition of structured activities is also consistent with past studies of structured leisure time, which have excluded time spent in school (e.g., Meeks and Mauldin, 1990; Larson and Verma, 1999; Mahoney and Stattin, 2000; Hofferth and Sandberg, 2001b; Fletcher et al., 2003; Osgood et al., 2005).

^2^Hofferth and Sandberg (2001b) separately identify reading, studying, and television watching as learning activities. However, we have classified reading and television as less-structured time, and studying as structured time, in keeping with other studies (e.g., Meeks and Mauldin, 1990; Eccles and Barber, 1999; Fletcher et al., 2003).

^3^Forward digit span tasks (where children repeat numbers in the order they are presented by an experimenter) primarily index storage capacity, rather than combined storage and processing capacity, and therefore do not serve as a reliable measure of EF (Daneman and Merikle, 1996; Engle et al., 1999).

^4^Aggregate within-measure scores were z-scored, then summed to create cross-measure composites.

^5^Social and enrichment events included only prior-week reporting, as these were not adequately identified in the annual less-structured time measure, which included only general activities (e.g., playing outdoor with friends) that could occur in many contexts.

^6^Here and in the following analysis, we also excluded all reading from our typical-activities measure, because this measure did not discriminate between reading alone and reading with others.

^7^This finding was not driven by a negative correlation between composite time in structured activities and time in less-structured activities. The less-structured and structured time composites were not significantly related (*p* > 0.8).

^8^Although it is not a targeted measure of conflict resolution, overall accuracy across all trials on the Flanker task has also been tested in prior intervention work with children (Rueda et al., 2005; Fisher et al., 2011; Röthlisberger et al., 2012), and is what improved in two prior intervention studies targeting EF in this age group (Fisher et al., 2011; Röthlisberger et al., 2012). Overall accuracy did improve with age in our sample [η^2^_*p*_ = 0.16; *F*_(1, 47)_ = 4.67; *p* < 0.04], but was not predicted by any other variables (*p*'s > 0.15).

^9^In separate analyses, we investigated whether the completeness of parent reporting of child time influenced observed relationships between child time use and EFs. For example, if parents who left fewer cells blank in the time use survey had children with higher self-directed EF, this could have contributed to the observed correlation between less-structured time and self-directed EF, since parents who left fewer cells blank might report more time in less-structured activities. However, completeness of reported time use did not affect the results: it showed no relationship with any aspect of EF performance (verbal fluency, AX-CPT, and Flanker *p*'s > 0.3), and controlling for it did not change whether or not any findings were significant.

^10^Recent less-structured experiences also predict self-directed EF when controlling for parent-reported typicality of the prior week [η^2^_*p*_ = 0.02; *F*_(1, 57)_ = 9.12; *p*<0.004; M_typ_ = 4; *SD* = 2.05; range = 1–7], and there is no interaction between less-structured experiences and typicality in predicting self-directed EF (*p* > 0.8). These findings might suggest that the prior week's experience is predictive separate from the extent to which it reflects typical/distal experiences. However, this interpretation rests on the validity and sensitivity of the typicality measure, which is unknown. Parent-reported typicality is at least internally consistent with parent-reported time use. Specifically, recent less-structured experiences predicted typical/distal experiences when parent-reported typicality of the prior week was high [M_typ_ = 6; η^2^_*p*_ = 0.03; *F*_(1, 60)_ = 5.81; *p* < 0.02], but not when typicality of the prior week was low (M_*typ*_ = 2; *p* > 0.9), yielding a marginally significant interaction [M_typ_ = 2; η^2^_*p*_ = 0.04; *F*_(1, 60)_ = 2.92; *p* < 0.093].

^11^For example, some EF-interventions have not improved performance on the Flanker task in this age group (Rueda et al., 2005, 2012; see also Diamond et al., 2007, which introduced switching demands that did show effects of intervention, and included only incongruent trials so that a standard conflict score could not be computed). The Flanker task can be sensitive to minor variations in stimulus parameters (Paquet, 2001) and intervention dosage in adults (Liu-Ambrose et al., 2012). Failures to find effects of interventions have also been attributed in part to the task's sensitivity to practice effects in pre-post measure designs (as discussed in Rueda et al., 2012), which are not an issue in the present study.

^12^We also note that there is ongoing debate regarding the inappropriateness of IQ as a control in models of cognitive processes (Dennis et al., 2009).
